# Dynamic Schwarz Meta‐Foams: Customizable Solutions for Environmental Noise Reduction

**DOI:** 10.1002/advs.202402872

**Published:** 2024-07-01

**Authors:** Daniel Saatchi, Saewoong Oh, Hyunjoon Yoo, Ji‐Seok Kim, Myung‐Joon Lee, Mannan Khan, Bernd Wicklein, Manmatha Mahato, Il‐Kwon Oh

**Affiliations:** ^1^ National Creative Research Initiative for Functionally Antagonistic Nano‐Engineering Department of Mechanical Engineering Korea Advanced Institute of Science and Technology (KAIST) 291 Daehak‐ro, Yuseong‐gu Daejeon 34141 Republic of Korea; ^2^ Materials Science Institute of Madrid (ICMM) Consejo Superior de Investigaciones Científicas (CSIC) Madrid 28049 Spain

**Keywords:** environment, meta‐foam, noise, soft metamaterial, tunable

## Abstract

In an era marked by increasing environmental challenges affecting human well‐being, traditional acoustic materials struggle to effectively handle the diverse and multi‐frequency nature of harmful environmental noises. This has spurred a demand for innovative acoustic metamaterial solutions by utilizing sustainable design strategies. This research introduces tunable Schwarz metamaterial capable of transforming into a soft meta‐foam to solve the complex problems of varying environmental noises. This study primarily focuses on adjusting single to multiple sound‐blocking bandgaps mechanism using a multi‐layered approach, incorporating the Schwarz P‐type triply periodic minimal surface (TPMS) and its elective soft foam counterpart, known as tunable Schwarz meta‐foams (TSMF‐x). The tunable design parameters of the unit cell, multi‐layered TPMS, and soft programmable TSMF‐lichen version are comprehensively explored including a fire‐safety test. The results demonstrate these enhanced flame retardant meta‐foam families have the potential to be used for mid‐to‐high‐frequency environmental noises in industrial equipment and smart homes for sustainable architecture and environmental health applications.

## Graphical abstract

The parametric design of an alternating unit cell of Schwarz primitive triply periodic minimal surface (TPMS) metamaterial can offer the capability to create both single tunable bandgap and multiple bandgaps. This versatility makes it suitable for addressing a wide range of varying environmental noises, spanning from industrial noise to the disruptive sounds experienced by building occupants in smart homes or workspaces.

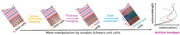

## 1 Introduction

In today's dynamic and bustling world dealing with different kinds of environmental problems,^[^ ^1^, ^2^, ^3^, ^4^, ^5^, ^6^, ^7^ ^]^ the issue of environmental noise has become an increasingly prevalent challenge, posing a significant environmental health problem in maintaining productively comfortable workspaces^[^ ^8^ ^]^ and quiet homes^[^ ^9^, ^10^ ^]^ to reduce additional stress even for sensitive babies in hospitals.^[^ ^11^ ^]^ Environmental noise can cause physical risks or mental health problems such as sleep difficulties,^[^ ^12^, ^13^, ^14^ ^]^ hyperacusis,^[^ ^15^ ^]^ hearing loss,^[^ ^16^, ^17^, ^18^ ^]^ misophonia,^[^ ^19^, ^20^ ^]^ and headaches^[^ ^21^, ^22^, ^23^, ^24^ ^]^ for building occupants. The sources of environmental noises are manifold,^[^ ^25^, ^26^, ^27^, ^28^ ^]^ spanning from the incessant hum of air conditioning units to the intermittent rumble of generators,^[^ ^29^, ^30^ ^]^ the operation of a construction site near the resident area,^[^ ^31^ ^]^ and even the harmonious varying mid‐to‐high frequencies yet often an intrusive deafening chorus of cicadas^[^ ^32^, ^33^, ^34^ ^]^ chirping during the summer noise^[^ ^35^, ^36^, ^37^ ^]^ months causing sleep problem. These auditory intrusions cover a wide range of frequencies, ranging from the low‐frequency droning^[^ ^38^, ^39^ ^]^ of rotational machinery^[^ ^30^, ^40^, ^41^, ^42^ ^]^ to the higher frequencies of various machinery equipment^[^ ^43^, ^44^, ^45^ ^]^ and the mid‐to‐high pitch of different cicadas buckling mechanism.^[^ ^32^, ^35^ ^]^ Quantifying these frequency ranges reveals a complex soundscape characterized by disparate tones.^[^ ^38^, ^41^, ^46^, ^47^, ^48^, ^49^, ^50^, ^51^, ^52^, ^53^, ^54^, ^55^, ^56^, ^57^ ^]^ The complexity of this issue underscores the urgent need for developing effective noise management solutions^[^ ^30^, ^31^, ^58^, ^59^, ^60^, ^61^, ^62^, ^63^ ^]^ and acoustic materials^[^ ^64^, ^65^, ^66^ ^]^ as a critical health concern.^[^ ^8^, ^9^, ^10^, ^49^, ^67^, ^68^, ^69^ ^]^

Traditional acoustic foam materials have faced difficulties in overcoming the complex challenges presented by environmental noise. Their effectiveness is limited because they struggle to manipulate sound waves efficiently across different frequency ranges, pinpoint problematic tonal frequencies, or achieve broad frequency range tuning.^[^ ^70^, ^71^, ^72^ ^]^ Recognizing these limitations, engineering scientists have turned their attention to the burgeoning field of acoustic metamaterials.^[^ ^39^, ^70^, ^73^, ^74^, ^75^, ^76^, ^77^, ^78^ ^]^ The innovative mechanical metamaterials offer a promising avenue for tailored acoustic wave manipulation, including the creation of bandgaps that selectively tune^[^ ^73^, ^79^ ^]^ and attenuate specific frequencies or target the problematic tonal frequency and short frequency range effectively by sound blocking. Recent advancements in acoustic metamaterials have even ventured into the realm of Triply Periodic Minimal Surfaces (TPMS), a geometric concept that presents a novel approach to controlling sound waves.^[^ ^80^, ^81^, ^82^ ^]^ TPMS‐based metamaterials have demonstrated remarkable potential in modulating acoustic waves at specific frequencies, heralding a new era in more efficient material science and acoustic metamaterial design.^[^ ^83^ ^]^ Previously, to improve the TPMS sound attenuation by additional sound absorption mechanism for non‐bandgap regions, the symbiosis mechanism concept was inspired by nature to turn Schwarz‐P TPMS metamaterial into symbiotic lichen‐Schwarz metamaterial (SLSM).^[^ ^84^ ^]^ Lichens become trending auxiliary natural materials for biophilic design and sustainable architecture. However, both Schwarz‐P TPMS and original SLSM in a previous study^[^ ^84^ ^]^ also have their limitation in providing a single narrow bandgap for sound‐blocking purposing which depends on the subwavelength of the scaled TPMS unit cell in SLSM.

To tackle the challenges of the limited narrow single bandgap of TPMS and improve acoustic material efficiency for environmental noise reduction, novel manipulation strategies to tune and widen the bandgap by providing multiple bandgaps solution involving advanced tunable design parameters of Schwarz‐P TPMS unit cell is proposed in this study. Then, hard‐to‐soft tunable TPMS metamaterials are offered for more efficient acoustic wave management by incremental alternation of unit cell characteristics through Schwarz's different design parameters. By alternating metamaterial design parameters of TPMS such as surface modulation, unit cell size, volume fractions, isotropic scaling, multi‐layering, and anisotropic scaling in one direction, high‐frequency environmental tonal noise can be targeted, and a broad frequency noise can be attenuated when the need for change arises. Next, a symbiotic version of tunable TPMS metamaterials is explored, and the concept of soft Schwarz meta‐foam families (TSMF‐x) is proposed. The calculations and experimental results concerning the advancement of TSMF‐lichen are further explored. Additionally, this study also aims to enhance partially eco‐friendly tunable acoustic metamaterials, addressing growing environmental issues like waste management carbon footprint, global warming, and heightened fire risks. Specifically, the study focused on ensuring safety features such as flame retardancy and fire resistance in building acoustic metamaterials, which were assessed through experimental measurements. The tunable and programmable TSMF‐lichen variants show promising potential in sustainable design, and adaptive architectural solutions, effectively reducing diverse industrial noises that are generated by equipment such as broadband‐noise generators. They can also be utilized for creating robotic shading systems in smart homes to reduce the impact of varying broadband environmental noise caused by cicada insects during the summer months.

## 2 Results and Discussion

**Figure 1:**
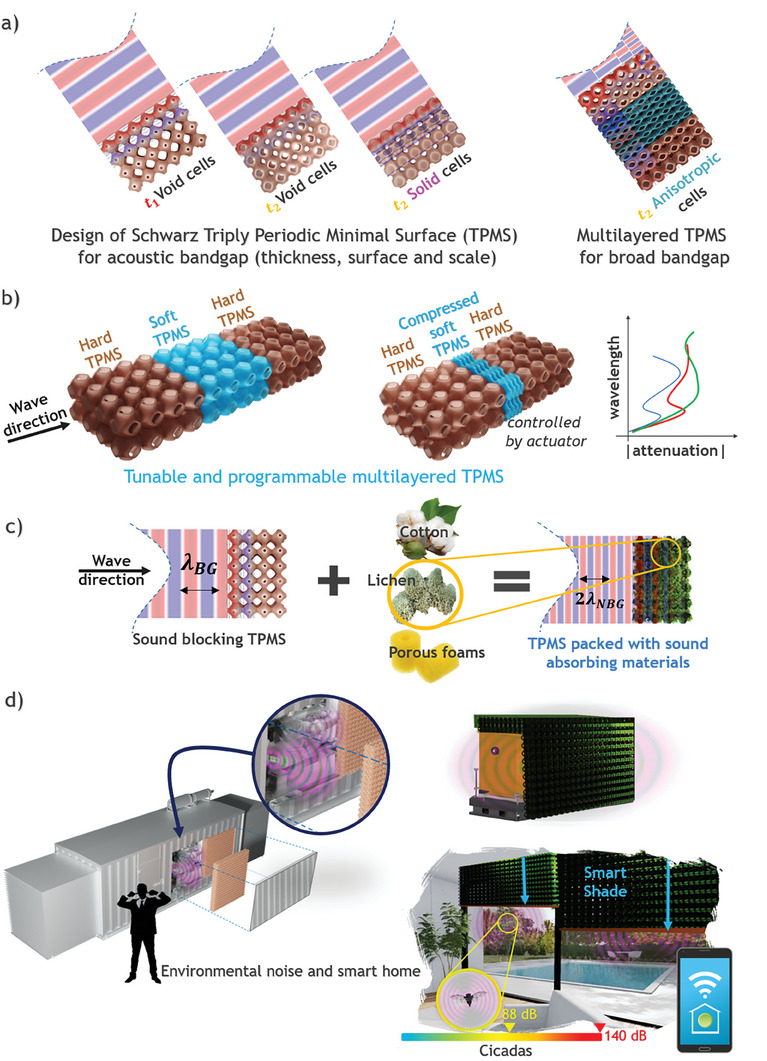
Schematic illustration of subwavelength manipulations for a) tunable single bandgap to multiple bandgaps by alternating design parameters of Schwarz‐P TPMS unit cell and layers. b) Assembly of hard TPMS and compressible soft TPMS. c) Conversion of tunable Schwarz TPMS metamaterial to tunable Schwarz meta‐foam (TSMF‐lichen). d) Applications for Industrial noisy equipment and cicada summer noise for robotic smart shades in smart homes.

The advanced and novel parametric designs of the tunable Schwarz meta‐foam (TSMF‐x) family are proposed for broad multiple bandgaps as illustrated in **Figure** **1**. The soft tunable TSMF‐lichen, which is TSMF filled with lichen, was designed for a specific target application in adaptive architecture with humidity control and flame‐retardant functionalities. The lichen can be changed with another foam with the same tunable Schwarz P‐type TPMS. Table S1 (Supporting Information) provides more insight into other foam candidates and future TSMF‐x families.

### 2.1 Concept of Tunable Schwarz Hard Metamaterial and Soft Meta‐Foam

The evolution of tunable Schwarz hard metamaterial to soft meta‐foam (TSMF‐x) involves a progression from a single bandgap to multiple bandgaps. This development is centered around the step‐by‐step modification of the Schwarz P‐type TPMS unit cell, with each alteration requiring specific attention. To develop TSMF‐x with broad multiple bandgaps, Figure 1a provides a comprehensive, step‐by‐step illustration of how the original Schwarz P‐type TPMS unit cell undergoes incremental alterations with different strategic design parameters, particularly by varying the parameter ***“t”*** in the TPMS equation from ***t***_1_ to ***t***_2_, leading to changes in the surface morphology of the unit cell. Subsequently, the thickness of the morphologically identical unit cell can be adjusted to transform void cells into solid cells by changing shell thickness, thereby achieving single bandgap tuning while the volume fraction (VF) of the unit cell changes. Next, by anisotropically scaling the unit cells in an X‐period direction only, it becomes possible to generate a multilayered TPMS to have multiple bandgaps within the same metamaterial, effectively blocking longitudinal sound waves across a range corresponding to the wavelength of the sound wave and the subwavelength of the scaled metamaterial. Both single bandgap and multiple bandgap design parameters can be integrated by assembling hard and soft TPMS components, as depicted in Figure 1b, enabling the creation of active versions of Schwarz P‐type TPMS. When the assembled hard TPMS parts compress the soft TPMS part, a secondary bandgap emerges alongside the original single bandgap in a reconfigured metamaterial, resulting in two multiple bandgaps in the X‐direction parallel to sound wave directional propagations, representing the simplest form of dynamic soft version of a tunable TPMS with multiple bandgaps.

To enhance sound attenuation in tunable TPMS, soft porous materials, such as biophilic foams like Reindeer lichen,^[^ ^84^, ^85^ ^]^ industrial foams like polyethylene, or natural fibers like cotton, are desirable. All of these foams with tunable Schwarz metamaterial are classified to be referred to as tunable Schwarz meta‐foam (TSMF‐x) families. The lichen plays a pivotal role in fabricating the SLSM and now tunable soft SLSM meta‐foam (TSMF‐lichen), as demonstrated in Figure 1c. However, it is worth noting that the fabrication process may pose challenges when working with different types of foams such as polyethylene, neoprene, cotton, or any other foams mentioned in Table S1 (Supporting Information). In one exemplar case, symbiotic lichen (fungi) is selected for TPMS, creating a symbiotic lichen‐Schwarz metamaterial (SLSM), which has been successfully fabricated previously by us.^[^ ^84^ ^]^ Derived from the basic version of SLSM, the TPMS undergoes a transformation into a tunable TPMS, which in turn makes the SLSM tunable as well. Both the tunable TPMS and TSMF‐lichen (SLSM) have practical applications in reducing mid‐to‐high‐frequency environmental noises with variable pitches. These applications include addressing diverse industrial noises, such as those emitted by noisy equipment like diesel generators,^[^ ^29^ ^]^ and contributing to the creation of robotic smart shades for smart homes. These smart shades act as a barrier against intrusive summer sounds, such as those made by insects like cicadas,^[^ ^32^ ^]^ preventing them from entering residential or commercial buildings, as illustrated in Figure 1d.

### 2.2 Design Parameters for Tunable Single Bandgap

**Figure 2:**
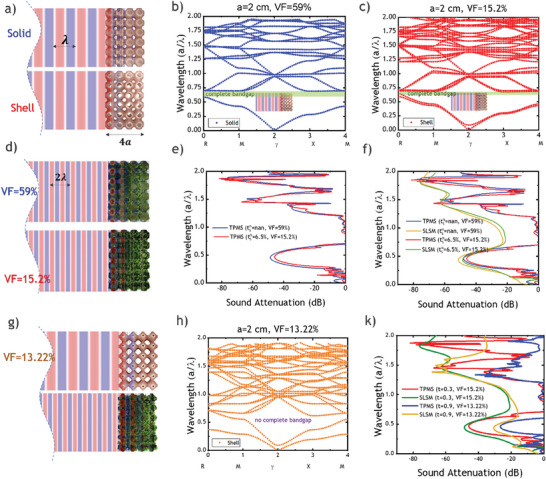
Tunable single bandgap design parameters. a) Volume fractions for identical solid TPMS versus shell TPMS with normalized shell thickness $t_{s}^{n}$. b) complete bandgap in solid version. c) Narrower complete bandgap in the shell version. d) Solid SLSM versus Shell SLSM. e) TPMS sound attenuation comparison. f) SLSM and TPMS sound attenuations. g) Changing parameter *“t”* for VF = 13.22%. h) Disappearance of complete bandgap for VF = 13.22%. k) sound attenuation comparison for all cases.

To comprehensively understand the design parameters of a single bandgap with scientific metrics, **Figure** **2** is presented to illustrate the influence of design parameter ***“t”*** behaving like an onion layer for surface morphology alternation, normalized shell thickness, $t_{s}^{n}$, and volume fraction (VF) on the complete bandgap within the dispersion curves. The VF is intricately determined by the values of parameter “***t***” and normalized shell thickness “$t_{s}^{n}$”. As depicted in Figure 2a, the variation in normalized shell thickness is investigated in detail as shown in Figure S1 (Supporting Information), while keeping the parameter “*t*” constant, thereby distinguishing between solid and void versions. Moving on to Figure 2b, the dispersion curve showcases a complete bandgap for the solid version with a VF of 59%, along with Figure 2c, which represents the void version with a VF of 15.2% and a slightly narrower complete bandgap. Subsequently, Figure 2d illustrates the conversion of TPMS into SLSM with the addition of lichen, presenting a volume fraction of lichen (VFL) at ≈41% for VF of 59% solid version and VFL of 84.8% for the 15.2% void version. The comparative sound attenuation of TPMS is provided in Figure 2e, where both the solid and void versions exhibit no sound attenuation out of the bandgap region. However, in Figure 2f, a comparison is made between SLSM and TPMS with different volume fractions and normalized shell thicknesses. It becomes evident that SLSM with a VF of 59% and VF of 15.2% have slightly superior performance showing remarkable attenuation by −20 dB compared to TPMS versions for non‐bandgap region near wavelength between 0.75 and 1.2 for in Figure 2f.

Next in Figure 2g, the identical normalized shell thickness **“** $t_{s}^{n}$ **”** is maintained constant while the surface morphology parameter ***“t”*** is changed this time, resulting in a shift in VF from 15.2% to 13.22% for the void version TPMS. This alteration is analyzed through the new dispersion curve for a VF of 13.22%, showcased in Figure 2h, leading to changes in the band structures and the disappearance of the narrow complete bandgap. Figure 2k provides insights into sound attenuation for a VF of 15.20% and VF of 13.22% for both TPMS and SLSM, revealing that the SLSM with a VF of 13.22% exhibits inferior performance compared to the SLSM with a VF of 15.2%. The narrow single bandgap can be tuned upward or downward by adjusting the size of the Schwarz unit cell **“*a*”**, which corresponds to the subwavelength of the metamaterial interacting with the wavelength **“*λ*”** of the incident sound wave. Consequently, the frequencies in the dispersion graphs and sound attenuation plots in Figure 2 are normalized based on “***a/λ***” to elucidate the relationship between the wavelength of the interacting sound wave and the subwavelength of the TPMS/SLSM metamaterials. To address the issue of the narrow bandwidth of the single tunable complete bandgap, the subsequent section explains multiple bandgap design parameters with metrics to broaden the overall bandwidth of the bandgap regions, thereby effectively blocking a wider range of sound wave frequencies for sound attenuation purposes, which is not achieved by tunable single bandgap.

### 2.3 Novel Design Parameters for Tunable Multiple Bandgaps

**Figure 3:**
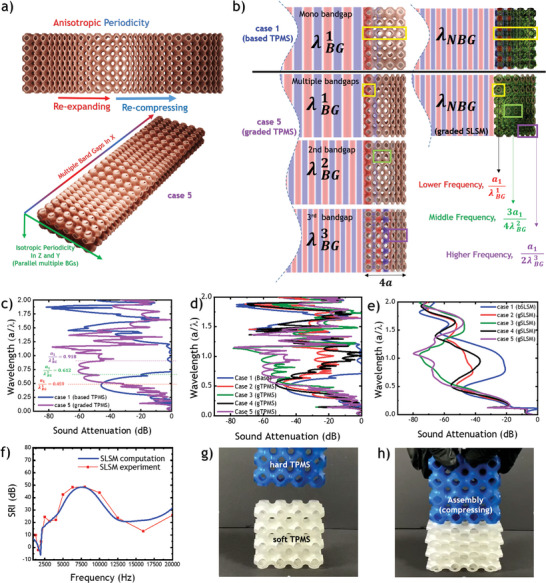
Multiple bandgaps formation by a) graded anisotropically scaled multi‐layered TPMS b) Multiple bandgaps subwavelengths corresponding to unit cells. c) Sound attenuation comparison between based TPMS and graded TPMS (gTPMS). d) Multiple combination comparisons for different graded TPMS e) Graded SLSM (gSLSM); TSMF‐lichen versions. f) computation and experimental comparison for SLSM. g) Assembly of hard TPMS and soft TPMS. h) Compressed soft TPMS by hard TPMS.

To understand the concept of tunable multiple bandgaps, **Figure** **3**a presents a schematic illustration of anisotropic periodicity, highlighting a novel aspect of this study. This illustration shows the formation of a hybrid metamaterial in the X‐direction, which combines aperiodic and periodic arrays using multi‐layered TPMS. This configuration results in the creation of multiple bandgaps in the X‐direction while maintaining a single bandgap in the Y and Z directions. Consequently, multiple frequencies and subwavelength structures can be achieved through customization. Figure 3b further elaborates on the distinction between a periodic TPMS array and an aperiodic‐graded TPMS (gTPMS) array with anisotropic cells, exemplified by case 5. It visually compares how wavelengths and subwavelengths are interrelated for longitudinal sound waves, highlighting the formation of multiple bandgaps. The fifth case in Figure 3b, in particular, features an original unit cell that mirrors the single complete bandgap of a base case at lower frequencies. However, the second and third cells have narrower original complete bandgaps and introduce a new secondary middle‐frequency bandgap. The third, fourth, and sixth cells are identical, representing half of the original cell and providing a third, higher‐frequency partial bandgap. Consequently, the fifth case boasts three overall multiple bandgaps shown in Figure 3b. Cases 1 to 4 are also schematically presented in the supporting information, along with the geometrical equations detailing their design parameters formation through manipulation of the multilayered TPMS equation and further dispersion graphs in Figures S2 and S3 (Supporting Information). The accompanying figures illustrate the relationship between data science and FEM data visualization in programming the dynamic version of the novel Schwarz meta‐foam. Anisotropic scaling of the unit cell alters the subwavelength bandgaps and band structures, allowing for the targeting of different frequencies. Figure 3c illustrates the superior sound attenuation of the fifth case (case 5) in comparison to the base case (case 1) at high frequencies, albeit showing slight inferiority within the original bandgap region. The reduced performance at lower frequencies is attributed to the lower number of original unit cells in the fifth case, which has been reduced from four to one per the same length of metamaterial. The design constraint of the fixed length prevents the metamaterial from becoming thicker while delivering moderate sound attenuation performance within a wider bandgap region by multiple bandgaps. The fixed length and arbitrary length equations are presented in Figure S4 (Supporting Information) to understand the concept better visually. More comprehensive comparisons among the based design case of TPMS (case 1) and its graded TPMS derivatives (case 2–5) are provided in Figure 3d for sound attenuations. Notably, the fifth case exhibits the best performance, owing to the combination of multiple bandgaps. Figures S5–S7 (Supporting Information) are provided in supporting information for sound propagation visualization in multilayered graded TPMS for better analysis. Subsequently, all TPMS cases are transformed into their graded SLSM (gSLSM) derivatives for further comparison of sound attenuation improvements, as demonstrated in Figure 3e. Once again, it becomes evident that the graded multilayer SLSM in the fifth case offers superior sound attenuation performance over a wider bandwidth owing to multiple bandgaps, in comparison to the other cases. Another comparative analysis of sound attenuation across all cases for total wavelengths is further detailed in Table S2 (Supporting Information). This analysis highlights that case 5 demonstrates slightly superior performance compared to case 3 when evaluated using discrete integral to measure the area below the wavelength axis.

To validate the basic concept, a comparison is made between finite element method (FEM) computations and experimental results for SLSM, assessing the sound reduction index (SRI) method^[^ ^86^, ^87^, ^88^, ^89^ ^]^ in Figure 3f, Figures S8 and S9 (Supporting Information). Finally, for the dynamic soft version, in reality, Figure 3g portrays the assembly of hard TPMS in blue and soft TPMS in white. By pressing on with the hard TPMS, the soft TPMS undergoes compression, leading to anisotropic changes in the cells, as demonstrated in Figure 3h and Video S1 (Supporting Information). The soft TPMS and soft SLSM versions are presented in Videos S2 and S3 (Supporting Information), which can be found in the supporting information. These videos offer a visual demonstration of the behavior and characteristics of these soft TPMS metamaterials, further complementing the information provided in the text and supporting figures.

### 2.4 Flame Retardant Tunable Symbiotic Lichen Schwarz Metamaterial

**Figure 4:**
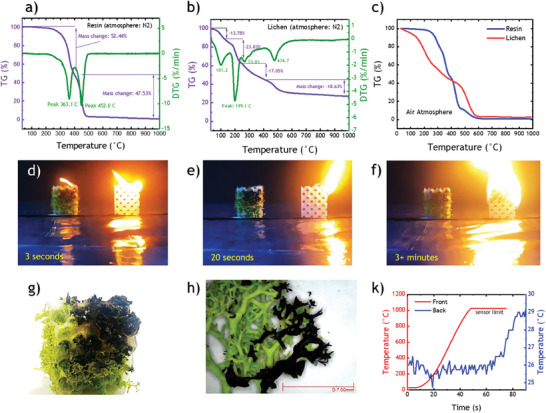
Thermogravimetric analysis results for a) solidified 3D printing resin and b) lichen in N_2_ atmosphere. c) TG curves of resin and lichen in air atmosphere. d) Flame retardancy test of SLSM (left, containing lichen) and TPMS (right, no lichen) with digital photographs taken after e) 3 s. f) after 20 s. g) Digital photograph of lichen in SLSM after self‐extinguished fire after 3 min. h) Optical microscope close of view of lichen. k) Thermocouple curves are taken on the front side and back side of the SLSM sample during a burn‐through test.

Sound absorbers and acoustic foams in buildings need to be flame‐resistant or flame‐retardant for safety. The obstacles in 3D printing acoustic metamaterials encompass factors such as cost and the need for tolerance, especially concerning flammable materials like the resin utilized in the process. Conventional polymer‐based TPMS with good tolerance is usually 3D printed by stereolithography lasering (SLA) technology. In SLA 3D printing, the photopolymerized resins are highly flammable, posing a significant challenge for this novel tunable acoustic metamaterial. One intriguing feature of Cladonia stellaris Reindeer lichen^[^ ^85^ ^]^ in tunable SLSM or TSMF‐lichen is its capacity to absorb relative humidity and water moisture. This makes the metastructure behave similarly to a flame retardant material because of moisture, as observed in **Figure** **4**. The thermogravimetric (TG) analysis results for the photopolymerized and solidified resin under N_2_ atmosphere are shown in Figure 4a. This resin is a mixture of methacrylic acid esters and photoinitiators ^[^ ^90^ ^]^ with similarities to other acrylic acid resins concerning thermal properties.^[^ ^91^, ^92^ ^]^ The thermal decomposition of the resin starts with a relatively steep decay following the onset temperature at 327 °C and the complete degradation at 500 °C. The first decomposition step centered at 363 °C (differential thermogravimetry curve) can be ascribed to the volatilization of low‐weight molecules and depolymerization of the acrylate chains, while the second step involves the degradation of the condensed, anhydrous fragments, possibly enhanced by the incorporated photocatalytic components. The associated mass losses are 52% and 48% (TG curve) leaving a residual mass of almost zero, which demonstrates that the resin material is consumed similarly to a fuel. On the other hand, the TGA curves of lichen in N_2_ atmosphere (Figure 4b) show the removal of physisorbed water (14% centered at 101 °C). Further mass change steps are centered at ≈199, 264, and 475 °C, which can be attributed to the volatilization of small molecules (CO_2_, H_2_O, CH_3_OH, etc.), continued depolymerization of the lichen biomass and condensation reactions of the anhydrous ring structures resulting in carbon‐rich char of high thermal stability.^[^ ^93^ ^]^ The TG graph shows that lichen is relatively stable at temperatures above 500 °C with a residual mass of 30% at 1000 °C. The thermal stability of lichen and resin was also investigated in air. Figure 4c reveals that lichen starts to degrade at lower temperatures (148 °C) than the resin (256 °C) but offers a higher resistance to char combustion at elevated temperatures (≈490 °C).

Next, three photo strips from Video S4 (Supporting Information) demonstrate the flame retardant properties of both TPMS and SLSM samples, with both beginning to burn when exposed to the flame, as depicted in Figure 4d. After a few seconds, the SLSM flame is extinguished, while the fire in TPMS propagates violently, as shown in Figure 4e. Infrared images of the burning materials are presented in Figure S10 (Supporting Information). The thermal camera is used to visualize how the heat is reduced after fire extinguishing, which may not be visible to the naked eye camera videos. The remarkable self‐extinguishing behavior of SLSM could be explained by the TGA results above. The release of water (and other noncombustible volatiles like CO_2_) from lichen at low temperatures below the onset of the thermal degradation of the resin possibly suppresses the decomposition and combustion process by cooling down the material, diluting the evolving combustible gases from the resin and even scavenging radicals. Figure 4g shows the scorched lichen and resin cells of SLSM confined locally and clearly demonstrates how lichen prevented the propagation of the flame. Partially smoldered lichen is observed under an optical light microscope showing black, charred ramifications that still maintain their morphology, highlighting again the excellent flame‐retardant behavior of lichen (Figure 4h). Further fire safety tests were performed by conducting a burn‐through test. Herein, two thermocouple sensors were installed on the front and back sides of the SLSM sample to monitor the temperature rise while a torch flame impinged on the front side. Detailed information about the thermocouple experiment is available in Figure S11 (Supporting Information) along with Video S5 (Supporting Information) for the burn‐through test. Figure 4k shows the two temperature curves from either side with a remarkable temperature drop of approx. 1000 °C across the 4 cm thick SLSM panel. Importantly, the back side showed only minute signs of smoldering (Figure S11b,c, Supporting Information) underlining the high resistance to fire. In summary, thanks to lichen, the resin‐based SLSM demonstrates a promising level of fire safety potentially interesting for building and equipment applications, especially in comparison to resin‐based/polymer‐based TPMS. In the next section, the tunable SLSM demo applications for smart home building and miscellaneous equipment are presented.

### 2.5 Varying Environmental Noises and Smart Home Applications

The proposed tunable and programmable TPMS, SLSM, and TSMF‐lichen have the potential to reduce mid‐to‐high frequency environmental noises with variations, which can originate from industrial or natural noise sources. For instance, noisy equipment like chillers,^[^ ^42^, ^94^ ^]^ generators,^[^ ^29^ ^]^ motors, compressors, pumps, or gas turbines produces a wide range of mid‐to‐high frequency noises due to varying operational speeds and revolutions per minute (RPM) required for tasks such as electricity generation or heating. The tunable TPMS and SLSM can be applied to shield this equipment by targeting specific operational frequencies tuned by the bandgap.

**Figure 5:**
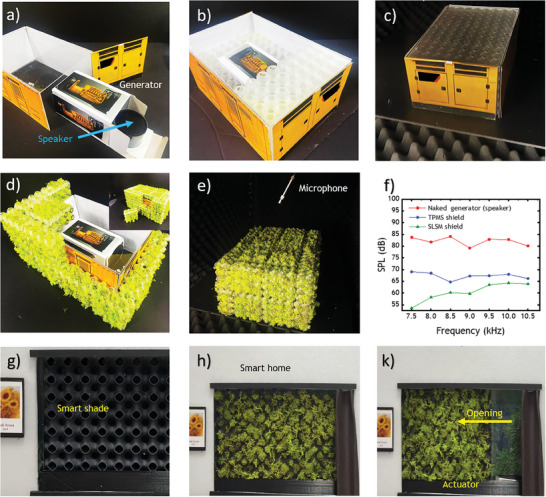
Application for industrial noise and summer noise. a) Speaker as a miniature generator. b) TPMS layer around diesel generator. c) Diesel generator container d) SLSM shielded container. e) Microphone position. f) Sound pressure level measurement comparison. g) soft TPMS smart shade and h) soft SLSM (TSMF‐lichen) smart shade being k) opened with linear actuator for the smart home.

To illustrate the configuration at a smaller scale, a demonstration using a miniature diesel generator is provided in **Figure** **5**. A high‐frequency speaker, as shown in Figure 5a, simulates the varying tonal sound frequencies of the diesel generator, which ranges from 7 to 11 kHz due to RPM variations by 0.5 kHz intervals. The sound generation is tonal to match the target frequency for the sake of acoustic measurement. This speaker is enclosed within a generator container. The generator container is internally shielded by TPMS with partial to complete bandgap ranging from 7.5 to 10.5 kHz, as depicted in Figure 5b. This TPMS‐shielded generator is placed in an anechoic space, as shown in Figure 5c. Next, the generator container is shielded with TPSM externally, as seen in Figure 5d. A microphone simulating human hearing is positioned to measure loudness and sound pressure level reduction (SPL), as illustrated in Figure 5e. An SPL comparison is presented for the naked generator in the container, the internally TPMS‐shielded generator in the container, and the externally SLSM‐shielded generator container in Figure 5f. The SLSM reduces the sound more than TPMS by showing lower SPL and TPMS SPL is lower down the naked generator. It's important to note that the shielding criteria for the diesel generator have to consider the need for air intake and ventilation in real cases, which is not the area of concern for this miniature demo application.

Another outlook application of soft TPMS and dynamic soft SLSM (TSMF‐lichen) can be considered for robotic smart home appliances, such as smart shades designed to reduce noise levels caused by cicadas in the summer.^[^ ^34^, ^37^, ^95^ ^]^ Cicadas produce varying mid‐to‐high frequency noises to deafening levels ^[^ ^32^ ^]^ that can be disruptive to sleep and concentration in working spaces. The soft TPMS smart shade is presented in Figure 5g, along with the closed soft SLSM smart shade for windows shown in Figure 5h. A robotic actuator can move the soft SLSM (TSMF‐lichen) shades when sensing high‐decibel sounds from outside, such as cicadas and other insect noises, to either close or open the shades in a smart home, as demonstrated in Figure 5k and presented in Video S6 (Supporting Information) with another additional customizable dynamic TPMS version in Video S7 (Supporting Information) for better understanding in the supporting information of the paper.

## 3 Conclusion

In this study, a tunable Schwarz soft meta‐foam (TSMF‐x) is presented that offers the ability to diminish environmental noises with different excitation frequencies. This is achieved by employing a programmable and adaptable soft TPMS layer. The unit cell of the Schwarz P‐type TPMS is altered through an incremental approach strategically, varying design parameters like volume fractions, isotropic scaling, deterministic factor, and normalized shell thickness to tune a single bandgap in TPMS. Subsequently, a multilayered TPMS is proposed by anisotropic scaling of the Schwarz unit cell to create multiple bandgaps, effectively blocking a wider broadband range of sound wave frequencies for sound attenuation purposes. These multiple bandgaps correspond to different subwavelength changes in new TPMS metamaterial and TSMF‐lichen, as detailed through in‐depth acoustic analysis. Then, a soft TPMS version is developed to complement the hard TPMS, enabling the tunable actuation of TPMS, SLSM, and TSMF‐x. While various foams can be used in TSMF‐x, lichen, a well‐established natural biophilic foam, was opted for in‐depth exploration and enabled fabrication of TSMF‐lichen in this study. Importantly, the moisture content in lichen within TSMF‐lichen serves as an effective fire retardant, delaying and extinguishing the highly flammable 3D‐printed TPMS. This was demonstrated through flame and thermocouple tests, ensuring enhanced fire safety. The applications of these adjustable metamaterials to meta‐foam extend to managing mid‐to‐high‐frequency environmental noises. This includes providing protection for various equipment such as generators, motors, pumps, and robotic shades in smart home setups.

## 4 Experimental Section

### Thermal Analysis

Thermogravimetric analysis (TGA) and differential thermal analysis (DTA) were performed in a nitrogen atmosphere (TG209 F1 Libra, Netzsch). Fire retardancy tests were carried out with a butane gas lab torch directed at metamaterial samples. In burn‐through tests, the same torch was used together with K‐type thermocouples (connectable to audrino kit) placed on both sides of the metamaterial panel reading the temperature. Thermographic images were recorded with a (Fluke) thermal imager. A K‐type thermocouple, comprised of chrome and alumel conductors, was employed, possessing a temperature range of −200–1260 °C (−326 to 2300 °F). To obtain temperature readings from the thermocouple, a thermocouple amplifier (MAX6675) was utilized. The amplifier incorporates a temperature sensor designed for measuring the temperature at the reference junction to amplify the tiny voltages. It was interfaced with the microcontroller through the SPI communication protocol and delivered the data at a 12‐bit resolution.

### Acoustic Analysis

TPMS geometries were generated using lab code that relies on new equations presented in this paper and previous studies. Blender software was employed to create the STL file for COMSOL simulation. The acoustic model was applied to TPMS for frequency domain analysis, covering both sound attenuation and dispersion curve calculations. The Delany‐Bazley‐Miki (DBM) porous acoustic model was utilized for SLSM sound attenuation. In the case of dispersion curve analysis, periodic boundary conditions were imposed for X, Y, and Z within the unit cell. For sound attenuation, the plane wave boundary condition was utilized. Sound attenuation and sound reduction index were computed following the methods outlined in the referenced paper. The Sound Reduction Index (SRI) method was conducted using Prosig hardware and software (model P8012).

## Conflict of Interest

The authors declare no conflict of interest.

## Author Contributions

D.S. performed conceptualization, design, computations, fabrication, experiment, demo, and writing the manuscript. S.O. co‐authorship and revision. H.Y. performed the TGA experiment. M.‐J.L. helped with metamaterial analysis. J.‐S.K. contributed to the design application of smart home and robotic actuator and demo providing. M.K. contributed to thermal sensors and programming with electronic kit programming for flame experiments. B.W. manuscript writing, revision, and contributed to TGA analysis and flame experiment. M.M contributed to manuscript writing, revision, and analysis. I.‐K.O as a principal investigator supervised and managed the collaborations, revision, and writing of the manuscript.

## Supporting information

Supporting Information

Supplemental Video 1

Supplemental Video 2

Supplemental Video 3

Supplemental Video 4

Supplemental Video 5

Supplemental Video 6

Supplemental Video 7

Supplemental Video 8

Supplemental Video 9

## Data Availability

The data that support the findings of this study are available from the corresponding author upon reasonable request.
