# Supplementary material for: Dynamic Schwarz Meta‐Foams: Customizable Solutions for Environmental Noise Reduction
Source: Adv Sci (Weinh). 2024 Jul 1;11(33):2402872. doi: 10.1002/advs.202402872 (PMC11434246; doi:10.1002/advs.202402872)
Supplement: Supplementary file 1 — Supporting Information [file ADVS-11-2402872-s003.docx]

Supporting Information

Dynamic Schwarz Meta-Foams: Customizable Solutions for Environmental Noise Reduction

Daniel Saatchi^^[[1]](#footnote-1)^^, Saewoong Oh^1^, Hyunjoon Yoo^1^, Ji-Seok Kim^1^, Myung-Joon Lee^1^, Mannan Khan^1^, Bernd Wicklein^2^, Manmatha Mahato^1^, Il-Kwon Oh^1^*

^1^ National Creative Research Initiative for Functionally Antagonistic Nano-Engineering, Department of Mechanical Engineering, Korea Advanced Institute of Science and Technology (KAIST), 291 Daehak-ro, Yuseong-gu, Daejeon 34141, Republic of Korea

^2^ Materials Science Institute of Madrid (ICMM), Consejo Superior de Investigaciones Científicas (CSIC), 28049 Madrid, Spain.

*Correspondence and requests for materials should be addressed to I.-K. Oh (E-mail: [ikoh@kaist.ac.kr](mailto:ikoh@kaist.ac.kr) )

Keywords: Tunable, Soft Metamaterial, Meta-Foam, Environment, Noise

**S1. Design Parameters, Humidity Results and Lichen Acoustic Modeling**

For parameters such as normalized shell thickness in **Equation 1**, volume fractions (VF) in **Equation 2**, volume fraction of lichen (VFL) in **Equation 3**, humidity results, and lichen acoustic modeling, the detailed sections pertaining to the design of SLSM are reported in earlier communication.^[84]^ The parameter "t" in TPMS equations, such as in **Equation 4** and **Video S8**, behaves in a manner analogous to an onion layer parameter for surface morphology. A brief summary of these parameters and their corresponding equations can be found in the **Supporting Information** provided here.

| The normalized thickness of the shell for the unit cell can be expressed as follows,  $t_{s}^{n}=\frac{t_{s}}{a}=\frac{size of shell thicknes (mm)}{size of unit cell (mm)}\times100\%$ | (1) |
| --- | --- |
| The normalized volume fraction (VF) with referenced cubic volume is,  ${VF}_{structure}=\frac{V_{3D print}}{a^{3}}\times100\%$ | (2) |
| The normalized volume fraction for lichen is,  ${VF}_{Lichen}=100-{VF}_{structure}$ | (3) |

The increase in normalized shell thickness, with its implications for geometry formation, operational 3D printing, and the ballooning violation of surface constraints, is schematically explained in **Figure S1** and **Video S9** for better understanding.


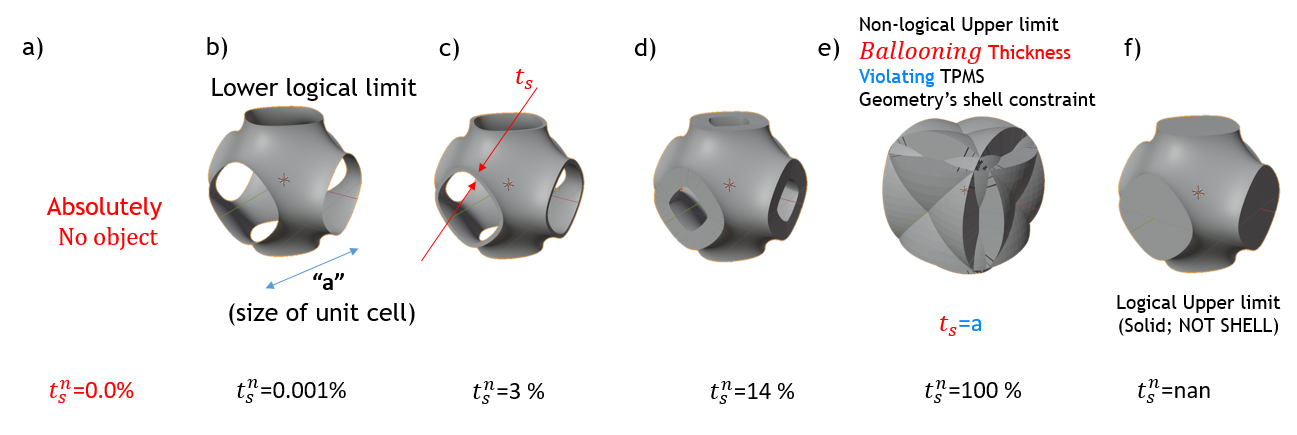


**Figure S1.** Normalized thickness of shell changes from a) no thickness, to b) lower logical limit, c) 3% thickness, d) 14% thickness, e) 100% thickness (non-logical upper limit), f) logical upper limit (solid; not shell anymore).

**Figure S1a** demonstrates that there will be no object if $\boldsymbol{t}_{\boldsymbol{s}}^{\boldsymbol{n}}$ is absolutely zero. **Figure S1e** illustrates the non-logical upper limit, where the thickness expands excessively, violating the surface constraints of the TPMS geometry's shell. **Figure S1f** depicts the logical upper limit for ***"***$\boldsymbol{t}_{\boldsymbol{s}}^{\boldsymbol{n}}\boldsymbol{=nan}$***"***, where there is no shell existence, resulting in a solid cell. The following section presents newly derived equations for advanced tunable single bandgap parameters and multiple tunable bandgap parameters as detailed in this paper.

**S2. Design Parameters of Anisotropic Schwarz Unit Cell**

In an anisotropic unit cell, a scaling unit cell is introduced in one direction parallel to the sound wave propagation direction. Sound waves are longitudinal waves, and this type of scaling is applied to the subwavelength of Schwarz P-type TPMS, SLSM, and TSMF-x structures. The anisotropic scaling in the unit cell is achieved by modifying the X-period in the trigonometric relationship within the geometrical mathematical formulation of the Schwarz P-type unit cell, as shown in the following format. The original formula is:

| $\cos\left( x \right)+\cos\left( y \right)+\cos\left( z \right)=t$  The anisotropic unit cell formula is expressed as, | (4) |
| --- | --- |
| $\cos\left( c_{gx}x \right)+\cos\left( y \right)+\cos\left( z \right)=t$  For compressed anisotropic scaling down (${AS}_{d}$) to shorter subwavelength, the following equation along its dispersion curves are presented in **Figure S2.** | (5) |
| $1<c_{gx}<4, {AS}_{d}=\frac{1}{c_{gx}}\times100$  And for stretched anisotropic scaling up (${AS}_{u}$) to bigger subwavelengths, the coefficient to change the periodicity is presented in the following equation along with **Figure S3** for better explanations of changes in band structures and bandgap changes. | (6) |
| $0.5<c_{gx}<1, {AS}_{u}=\frac{1}{c_{gx}}\times100$ | (7) |


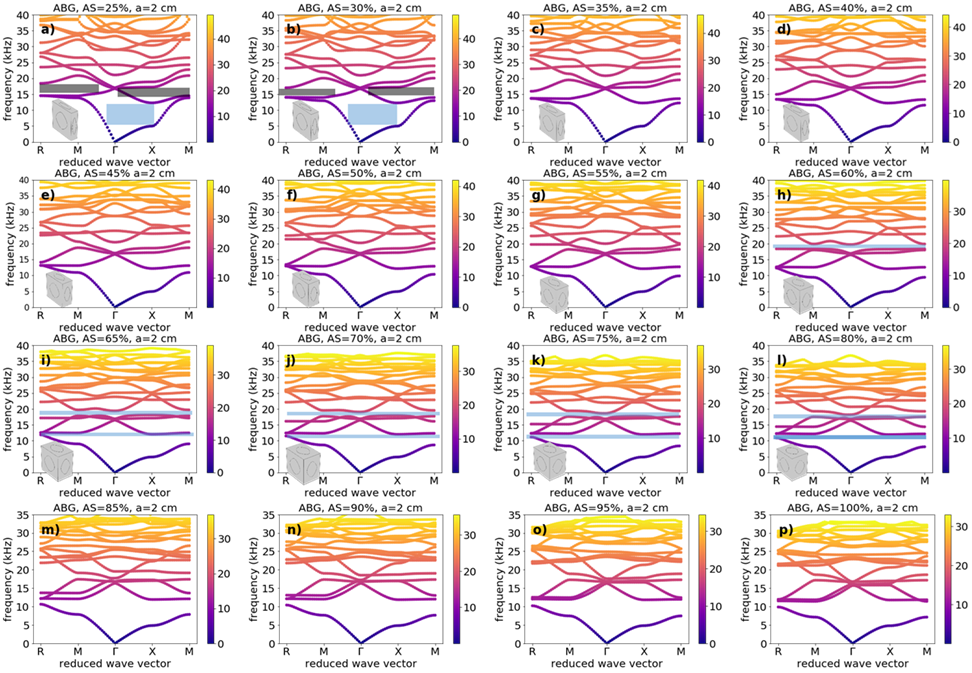


**Figure S2.** Multiple acoustic bandgap (ABG) for anisotropically scaled (AS) downed TPMS. Partial bandgaps at **a)** AS of 25%, **b)** AS of 30%, **c)** AS of 40%, **d)** AS of 45%, **f)** AS of 50%, and **g)** AS of 55%. **h)** The first complete bandgap at AS of 60%. Two complete bandgaps for **i)** AS of 65%, **j)** AS of 70%, **k)** AS of 75%, and **l)** AS of 80%. Disappearance of second bandgap for **m)** AS of 85%, **n)** AS of 90%, **o)** AS of 95%, **p)** AS of 100, the original unit cell


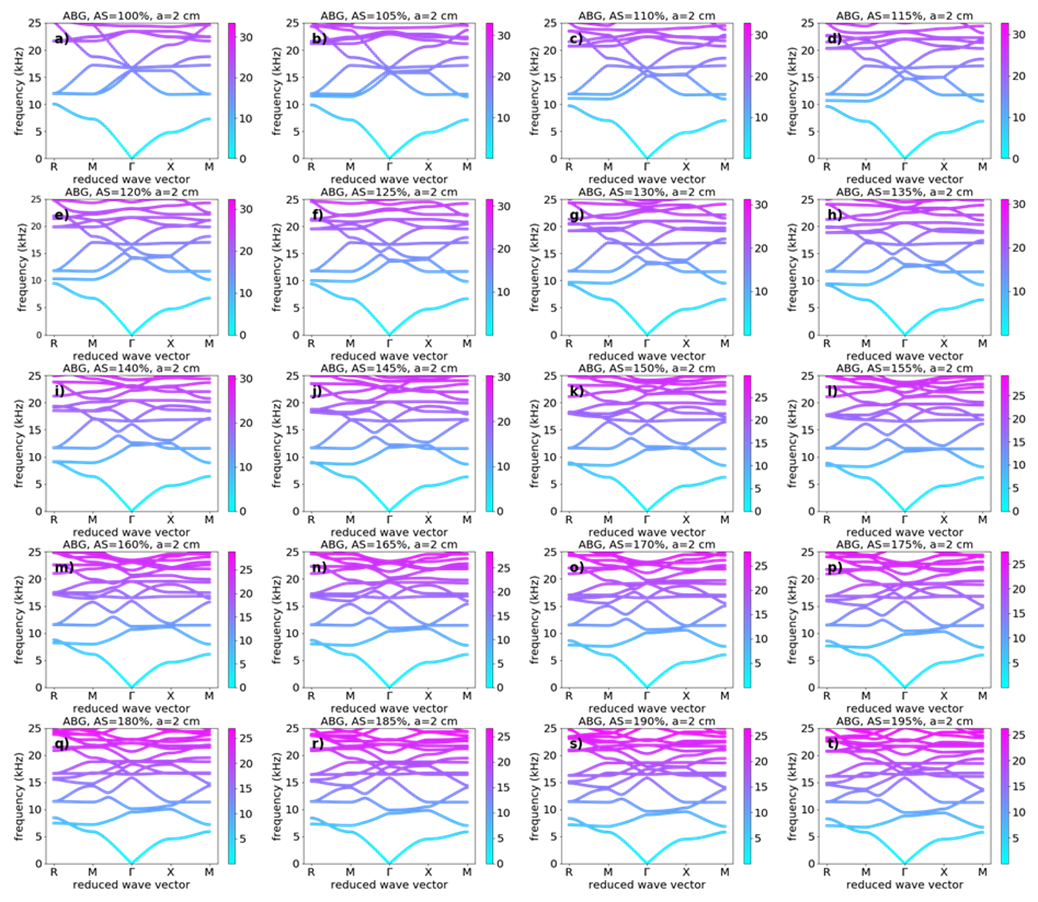


**Figure S3.** Acoustic bandgap (ABG) for Anistropically scaled (AS) up TPMS. Complete bandgaps become narrower for **a)** AS of 100%, **b)** AS of 105%, **c)** AS of 110%, **d)** AS of 115%, **e)** AS of 120%, **f)** AS of 125%, and **g)** AS of 130%. Partial bandgaps for **h)** AS of 135%, **i)** AS of 140%, **j)** AS of 145%, **k)** AS of 150%, **l)** AS of 155%, **m)** AS of 160%, **n)** AS of 165%, **o)** AS of 170%, **p)** AS of 175%, **q)** AS of 180%, **r)** AS of 185%, **s)** AS of 190%, **t)** AS of 195%.

**S3. Multiple bandgap combinations**

Multiple bandgaps can be achieved through a multilayered graded TPMS metamaterial by combining an anisotropically scaled-down (AS) unit cell of the Schwarz P-type. From the perspective of metamaterial thickness, it is possible to set a fixed length to create significantly broader sound-blocking bandgaps. This is advantageous for enhancing sound attenuation performance.

For example, **Figure S2** illustrates the selection of unit cell sizes with AS values of 100%, 75%, and 50%, all set to the fixed length "4a." Six combinations, ranging from Case 1 to Case 6, are formed. Case 1 represents the original design based on four original unit cells, as shown in **Figure S4a**. In Case 2, five-unit cells are used, comprising three original unit cells, one with an AS of 100%, and two half unit cells with an AS of 50%, as depicted in **Figure S4b.** Case 2 exhibits two different band structures: one complete bandgap for the original cell and one partial bandgap in the Г-X direction. Case 3 consists of six-unit cells, including two original cells with an AS of 100% and four half-cells with an AS of 50%, shown in **Figure S4c**. In Case 4, you can find two original unit cells with an AS of 100%, two third/quarter cells with an AS of 75%, and one half-cell with an AS of 50%, as shown in **Figure S4d**. **Figure S2k** demonstrates that AS of 75% yields two complete bandgaps, AS of 50% results in a partial bandgap in **Figure S2f**, and AS of 100% produces one complete bandgap for different subwavelengths of TPMS metamaterial. Additional combinations with AS values of 100%, 75%, and 50% are possible for Case 5, with six-unit cells shown in **Figure S4e**. Finally, for a partial bandgap with an AS of 50%, Case 6 is created with eight-unit cells, all at a fixed length of 4a, as shown in **Figure S4f**.

A few equations for this integer value can be mathematically derived per depth for length for a fixed length of 4a for case 1, where ***N*** is,

| $\sum_{i=1}^{N} a_{i}=Na, a_{i}=a$  For N=4, and fixed length of 4a for case 2, | (8) |
| --- | --- |
| $\sum_{i=1}^{4} a_{i}=4a$  For case 2 with AS of 100% and AS of 50%, | (9) |
| $\frac{{AS}_{100\%}}{100}\sum_{i=1}^{N-1} a_{i}+\frac{{AS}_{50\%}}{100}\sum_{i=N}^{N+1} a_{i}=Na, N>1$  For N=4, and fixed length of 4a for case 2, | (10) |
| $\sum_{i=1}^{3} a_{i}+\frac{1}{2}\sum_{i=4}^{5} a_{i}=4a, N=4$  For case 3, with another combination of AS of 100% and AS of 50% | (11) |
| $\frac{{AS}_{100\%}}{100}\sum_{i=1}^{N-2} a_{i}+\frac{{AS}_{50\%}}{100}\sum_{i=N-1}^{N+2} a_{i}=Na, N>2$  For N=4, and fixed length of 4a for case 3, | (12) |
| $\sum_{i=1}^{2} a_{i}+\frac{1}{2}\sum_{3}^{6} a_{i}=4a, N=4$  For case 4, with AS of 100%, AS of 75%, and AS of 50%, | (13) |
| $\frac{{AS}_{100\%}}{100}\sum_{i=1}^{N-2} a_{i}+\frac{{AS}_{75\%}}{100}\sum_{i=N-1}^{N} a_{i}+\frac{{AS}_{50\%}}{100}\sum_{i=N+1}^{N+1} a_{i}=Na, N>2$  For N=4, and fixed length of 4a for case 4, | (14) |
| $\sum_{i=1}^{2} a_{i}+\frac{3}{4}\sum_{i=3}^{4} a_{i}+\frac{1}{2}\sum_{i=5}^{5} a_{i}=4a, N=4$  For case 5, with AS of 100%, AS of 75%, and AS of 50%, | (15) |
| $\frac{{AS}_{100\%}}{100}\sum_{i=1}^{N-3} a_{i}+\frac{{AS}_{75\%}}{100}\sum_{i=N-2}^{N-1} a_{i}+\frac{{AS}_{50\%}}{100}\sum_{i=N}^{N+2} a_{i}=Na, N>3$  For N=4, and fixed length of 4a for case 5, | (16) |
| $\sum_{i=1}^{1} a_{i}+\frac{3}{4}\sum_{i=2}^{3} a_{i}+\frac{1}{2}\sum_{i=4}^{6} a_{i}=4a, N=4$  For case 6, with AS of 50% only, | (17) |
| $\frac{{AS}_{50\%}}{100}\sum_{i=1}^{2N} a_{i}=Na$  For N=4, and fixed length of 4a for case 6, | (18) |
| $\frac{1}{2}\sum_{i=1}^{8} a_{i}=4a$  For arbitrary lengths with multiple bandgaps, | (19) |
| $\frac{{AS}_{100\%}}{100}\sum_{i=1}^{m} a_{i}+\frac{{AS}_{75\%}}{100}\sum_{i=1}^{p} a_{i}+\frac{{AS}_{50\%}}{100}\sum_{i=1}^{q} a_{i}+\ldots=arbitary length$ | (20) |
| For instance, for case 5 in equation 17, the number for combination is:  $number of cells: m=1, p=2, q=3$ | (21) |


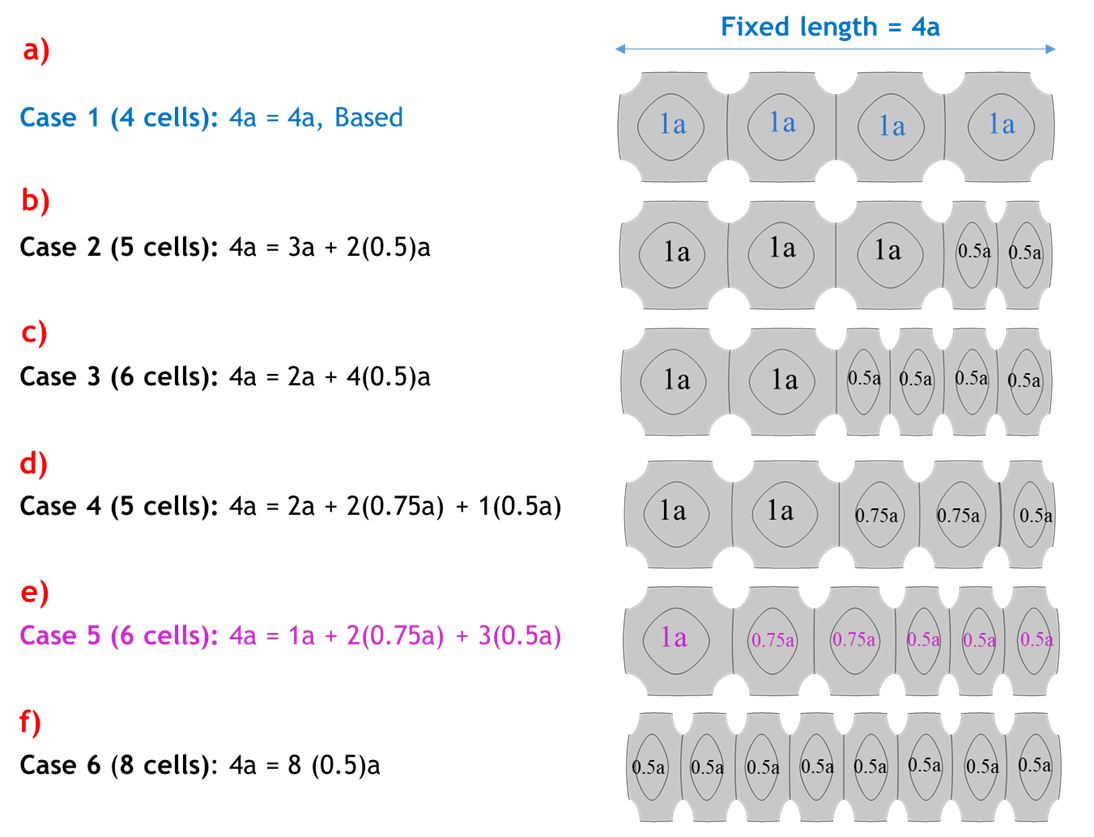


**Figure S4**. Multiple bandgap combinations per same fixed length for integer values. **a)** Single complete bandgap based case 1 with 4 identical unit cells with AS of 100%. **b)** Graded case 2 with 5 non-identical unit cells with AS of 100% and AS of 50%. **c)** Graded case 3 with 6 unit cells with AS of 100% and AS of 50%. **d)** Multiple complete bandgap with 5 graded unit cells with AS of 100%, AS of 75%, and AS of 50%. **e)** Multiple complete bandgaps with 6 graded unit cells for AS of 100%, AS of 75%, and AS of 50%. **f)** No complete bandgap with 6 graded identical unit cells with partial bandgaps only for AS of 50%.

Furthermore, the finite element method (FEM) results for combinations of multiple bandgaps are analyzed for Case 1 to Case 5, focusing on the acoustic pressure iso-surface (API), as shown in **Figure S5**, and the sound pressure level (SPL), as demonstrated in **Figure S6**. This analysis aims to understand the sound-blocking mechanisms associated with multiple bandgaps. It is evident from **Figure S5** that, for Case 1 and Case 4, the incident 15 kHz sound wave is not effectively blocked, while in the other cases, both 8 kHz and 15 kHz waves are effectively blocked. Similarly, in the SPL analysis, the sound pressure levels transition from red to green to blue for Case 2, Case 3, and Case 5, indicating a significant reduction in sound pressure for both 8 kHz and 15 kHz frequencies in the presence of multiple bandgaps.


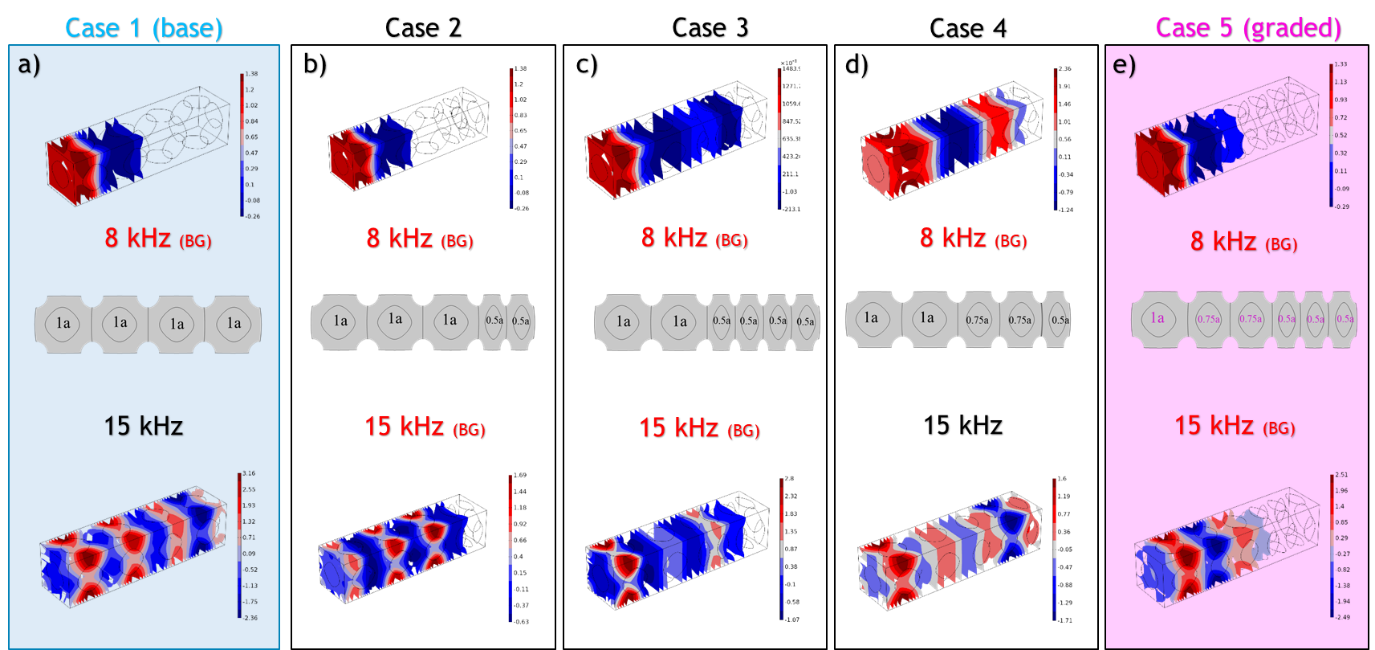


**Figure S5.** Multiple bandgaps combinations acoustic pressure iso-surface (API). **a)** Case 1 for single complete bandgap. **b)** Case 2 with multiple bandgaps. **c)** Case 3 with multiple complete bandgaps. **d)** Case 4 with multiple bandgaps. **e)** Case 5 with multiple bandgaps.

***
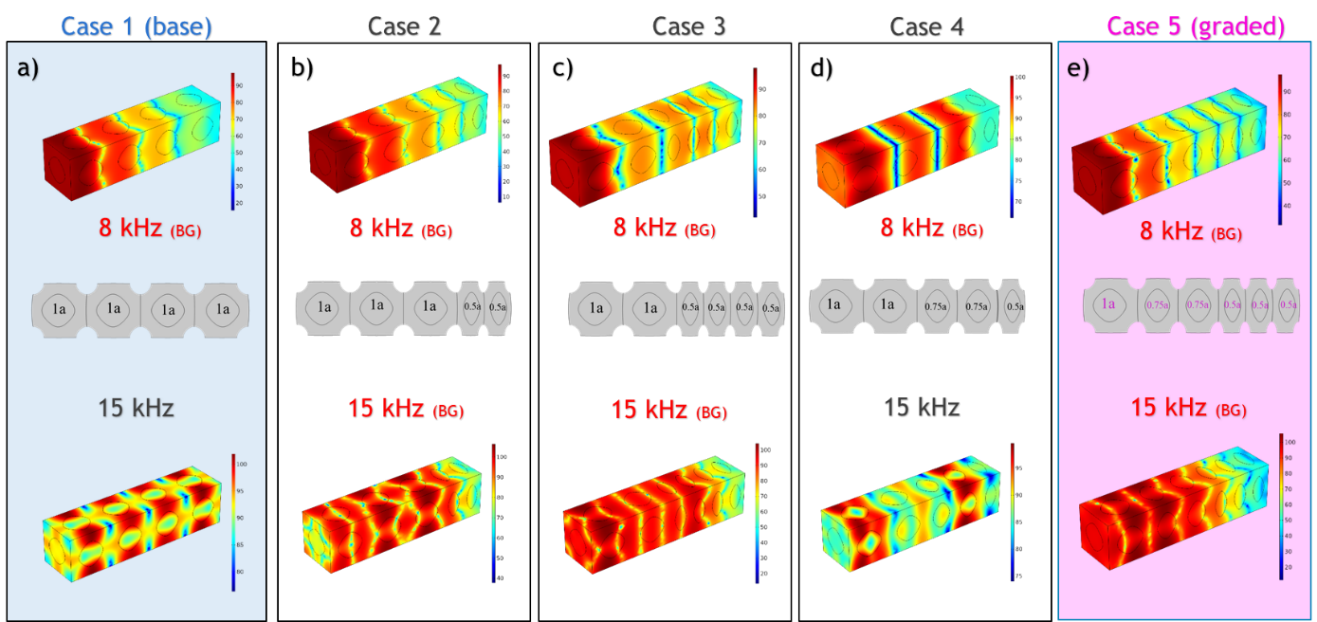
***

**Figure S6.** Multiple bandgaps combinations sound pressure level (SPL). **a)** Case 1 for single complete bandgap. **b)** Case 2 with multiple bandgaps. **c)** Case 3 with multiple complete bandgaps. **d)** Case 4 with multiple bandgaps. **e)** Case 5 with multiple bandgaps.

**
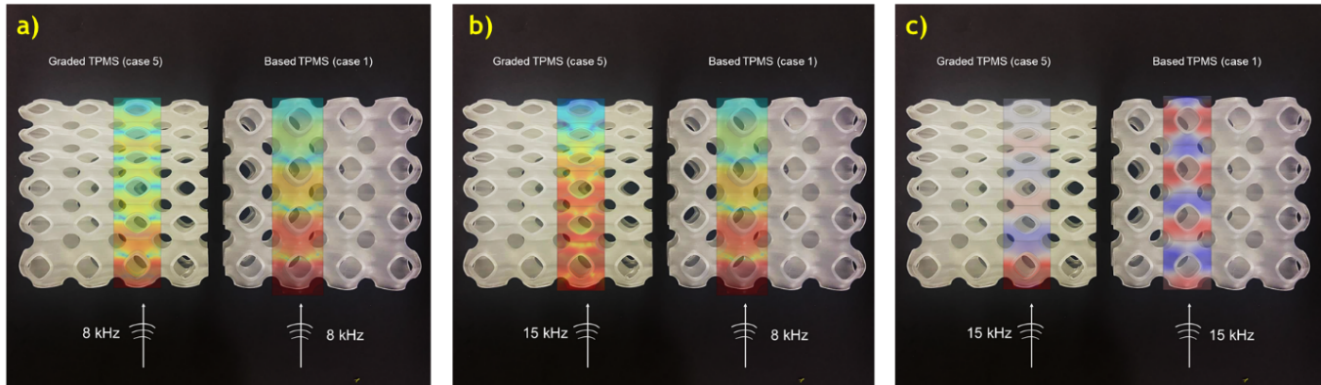
**

**Figure S7.** 3D printed panels. **a)** SPL of multiple bandgaps vs. single bandgap for 8 kHz. **b)** SPL of multiple bandgaps for 15 kHz vs. single bandgaps for 8 kHz. **c)** API of multiple bandgaps for 15 kHz vs. single bandgap for 15 kHz.

**S4. Acoustic Experiment**

The acoustic experiment is conducted using the Sound Reduction Index (SRI) method. **Figure S8** illustrates the setup, where the SLSM sample is positioned in the sample test area, as shown in **Figure S8a**, positioned between microphone 1 (the source) and receiving microphone 2, which is placed after the sample, as depicted in **Figure S8b**. Experimental measurements are carried out in one-third octave bands and then compared with the computational results for the SLSM.

**
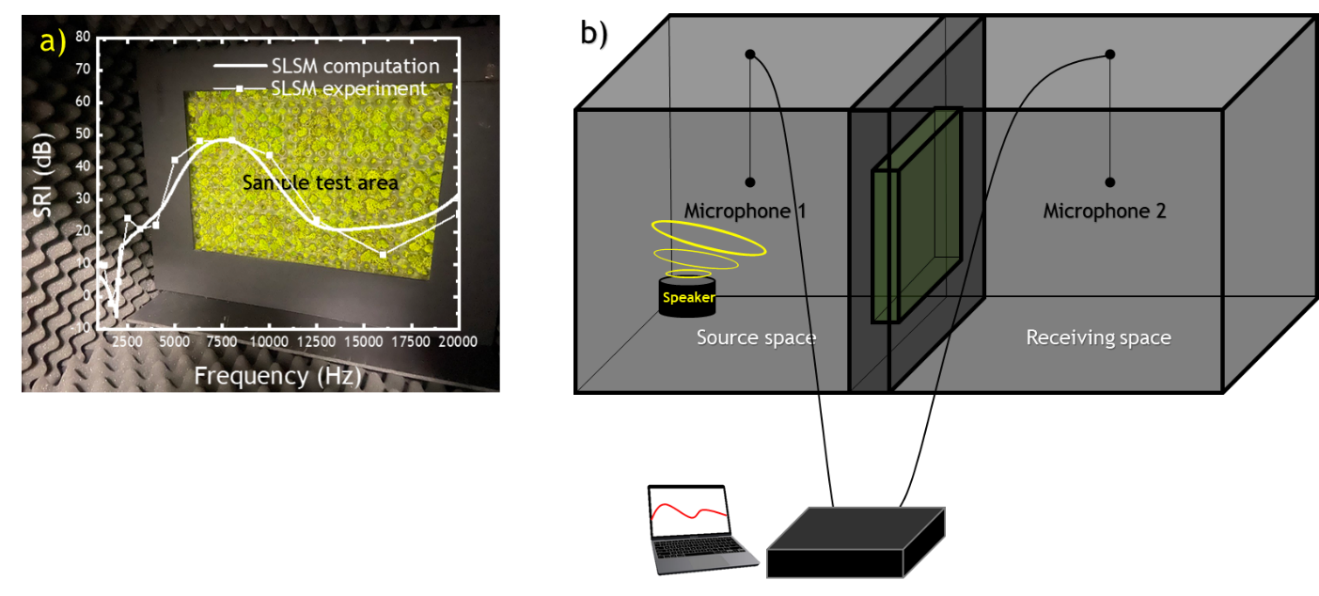
**

**Figure S8.** Sound reduction index acoustic experiment. **a)** SLSM sample test area in the middle. **b)** Speaker and microphone setups for source space and receiving space for the anechoic chamber.

Furthermore, a comparison between computational modeling and experimental data for lichen is presented in **Figure S9**. This figure illustrates the measurement of the sound absorption coefficient with an impedance tube and FEM for density, porosity factor, and flow resistivity factor,$R_{f}$, in porous acoustic modeling for the lichen, which serves as the natural foam for TSMF-lichen.


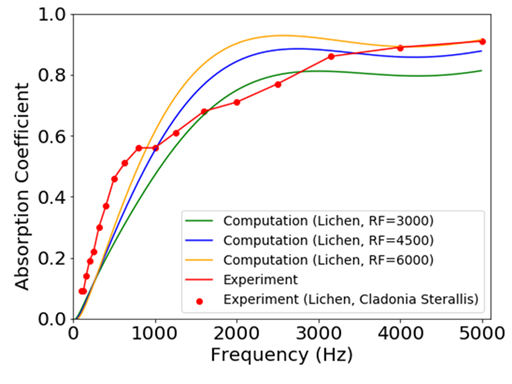


**Figure S9.** A comparison between computations and experiment for lichen sound absorption coefficient for porosity and flow resistivity effect.

**S5. Flame Retardant Experiment**

Flame retardant tests were conducted for both TPMS and SLSM, and these tests were recorded by a video camera and a thermal camera simultaneously, capturing a time-lapse sequence. The 3D SLA-printed resin used in both TPMS and SLSM is a highly flammable material that burns like fuel. While 3D SLA printing is one of the best methods for creating TPMS and SLSM structures, it does have a flammability drawback. The lichen incorporated into SLSM exhibits flame retardancy, primarily due to the presence of moisture in the lichen that delays the burning of SLSM and can even lead to self-extinguishing when the flame or fire is removed. **Figure S10** is provided to illustrate the comparison between infrared images and video images over time, showcasing how the TPMS sample ignites while the SLSM sample begins to self-extinguish and cool down.

**
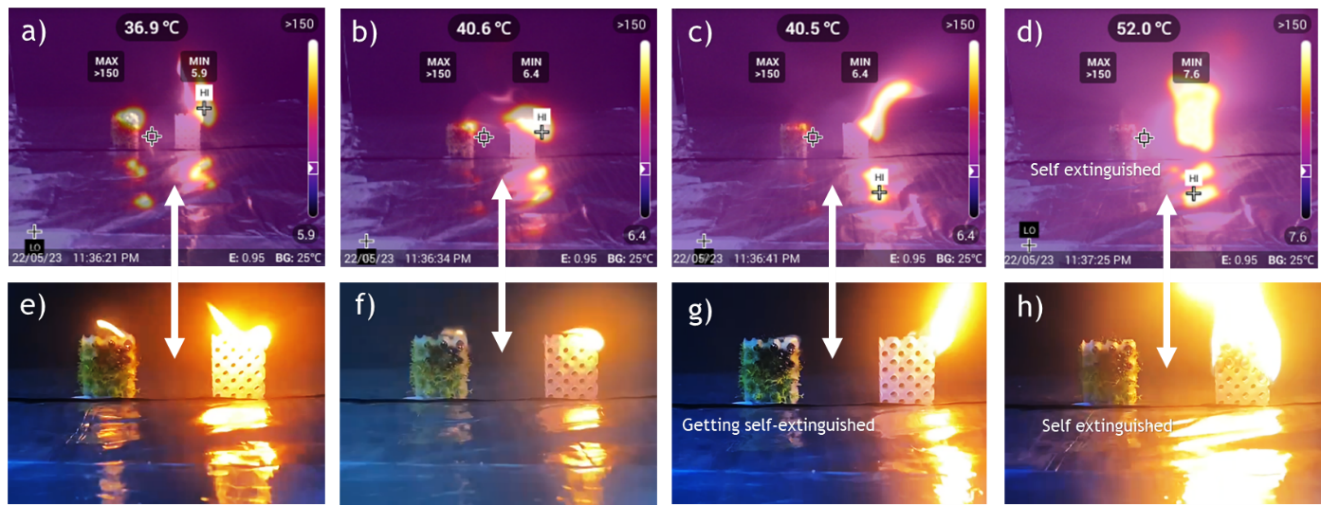
**

**Figure S10.** Paired Infrared camera with a real camera. The SLSM is getting self-extinguished over time from figures **a)** flame starts. **b)** TPMS flames up. **c)** TPMS flames become more while SLSM becomes is getting self-extinguished. d) SLSM self-extinguished shown in thermal camera. **e)** Real flame image. **f)** Smaller flame. g) No flame with the naked eye. **h)** Self-extinguished SLSM, but TPMS keeps getting burned.

**S6. Thermocouple Fire Experiment**

The thermocouple fire experiment was also conducted using a constant flame torch burning from one side of the sample to the other, as depicted in **Figure S11**. The first thermocouple sensor, located on the front side, measured the temperature on the side being exposed to the torch flame, as shown in **Figure S11a**. The second thermocouple sensor on the back side measured the temperature until the moment when the sample started burning in the middle, which is indicated by the appearance of smoke, as captured in **Figure S11b**. The black spot in **Figure S11c** represents the area that has been burned. A direct comparison between the front-side and back-side temperature measurements is presented in **Figure S11d** to show the lateral side.

**
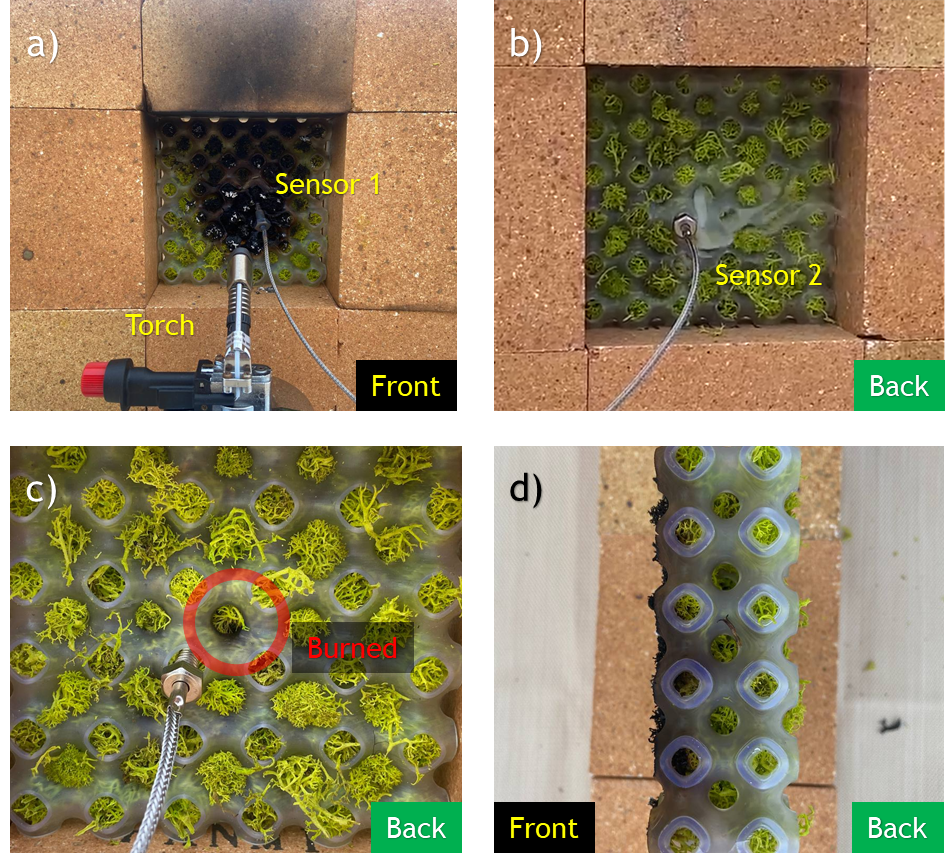
**

**Figure S11.** Thermocouple fire test. **a)** Front side with torch and sensor 1. **b)** Smokes in middle near sensor 2 in the backside. **c)** Burned spot in the middle of the back side. **d)** Lateral side comparison between the front and back sides of the sample.

**S7. Proposed TSMF-x family classification**

TSMF-x family comprises tunable Schwarz metamaterials with different foams, ranging from natural porous materials like reindeer lichen in soft SLSM to industrial foams such as neoprene or polyurethane foam, as indicated in **Table S1.** Each of these materials has its own set of limitations and challenges. For instance, the biophilic reindeer lichen is sensitive to relative humidity (RH), becoming dry and fragile when RH falls between 15% and 30%. This makes it challenging to maintain a stable dynamic TSMF-lichen, as it requires an RH environment above 30%-43%. Another natural biophilic option is cotton boll, which isn't RH-dependent but is more flammable due to its fibrous nature. Industrial foams like polyurethane, polyethylene, melamine polyester, and neoprene are stable candidates, yet their fabrication process poses challenges in combining the foams with Schwarz P-type 3D printing metamaterial. Consequently, future research on enhancing TSMF-x family with these foams presents an intriguing research topic and an avenue for exploring potential new applications and more efficient TSMF-x.

**Table S1.** Tunable Schwarz-p meta-foam family (TSMF-x)

| **Type of Foam** | **TSMF-x** | **Year made** | **Challenge** |
| --- | --- | --- | --- |
| Lichen (soft SLSM) | TSMF-lichen | 2023 | Dried lichen |
| Cotton bolls | TSMF-cotton | No | Highly flammable |
| Polyurethane | TSMF-polyurethane | No | Fabrication process |
| Polyethylene | TSMF-polyethylene | No | Fabrication process |
| Melamine Polyester | TSMF-melamine | No | Fabrication process |
| Neoprene | TSMF-neoprene | No | Fabrication process |

**S8. Programmable Acoustic Metamaterials for Wave Control System**

To comprehend the programmable soft TPMS metamaterial and programmable TSMF-lichen for sound wave control systems, **Figure S12** illustrates the process. First, the target noise frequency is measured by a microphone to ascertain the sound pressure level, determining whether it exceeds 85 decibels, the threshold for human hearing comfort according to OSHA standards. Subsequently, a sound control computing unit evaluates the available dataset for dispersion curves (**Figures S2 and S3**) and the bandgap database for corresponding aspect ratios (AS%). It then selects the optimal bandgap performance to determine the appropriate AS%.

The control computing unit commands the linear actuator to compress the soft TPMS to the corresponding AS%. The microphone sensor continuously monitors and measures the sound pressure level of the environment, providing input to the sound control computing unit. This unit adjusts the actuator for the programmable metamaterial or meta-foam version, aiming to achieve the best possible performance in noise reduction for smart shades and smart home window systems. Detailed explanations and additional context are provided in the Supporting Information.


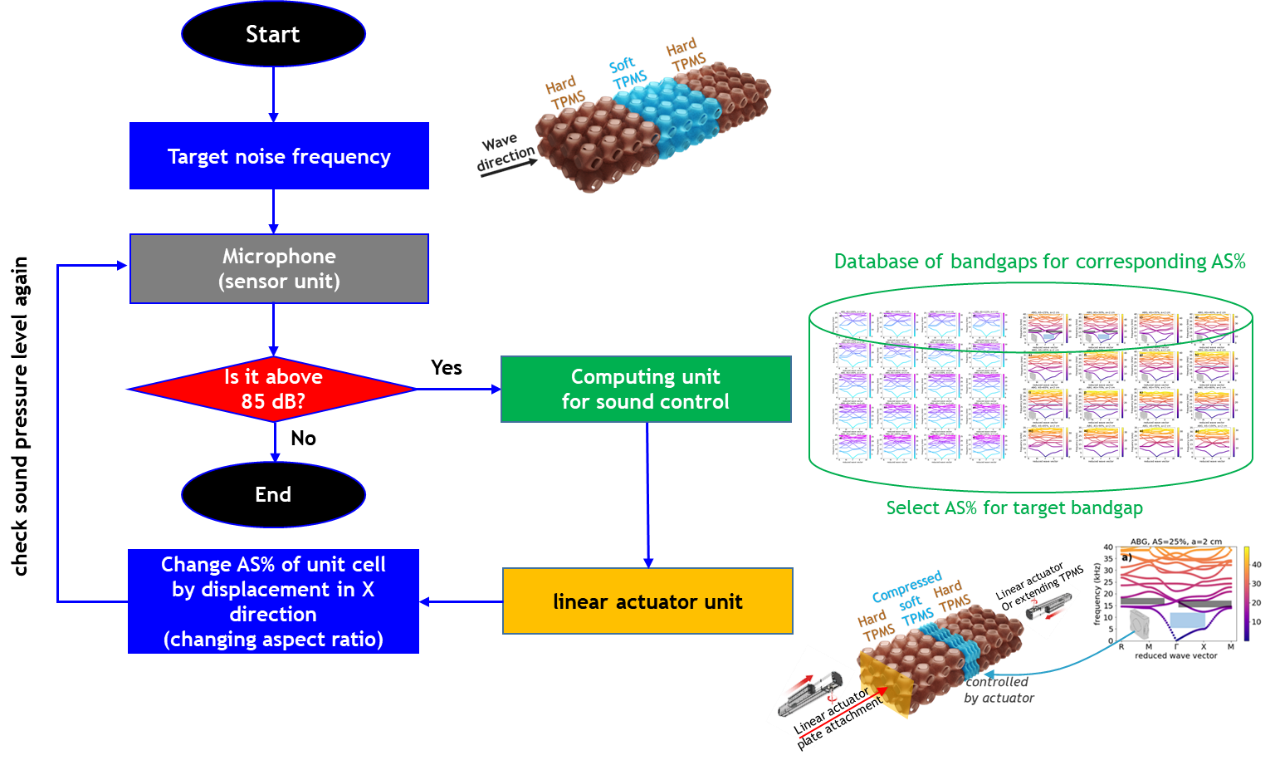


**Figure S12**. Workflow for programmable soft TPMS metamaterial inside meta-foam in wave control system with microphone sensor, frequency computing unit, and linear actuator.

As detailed and visualized in **Figures S2, S3,** and **S4**, anisotropic scaling alters the aspect ratio of the TPMS metamaterial unit cell, thereby influencing the band structure and causing shifts in bandgaps that affect the blocking of sound frequencies. Consequently, sound wave frequencies can be selectively targeted by adjusting the aspect ratio of the TPMS unit cell using the linear actuator in the programmable soft metamaterial and TSMF-lichen. This adjustment allows adaptation to the desired bandgap region through mechanically compressed displacement along the X-direction, changing the aspect ratio of the compressed cells. The implementation of a linear actuator and microphone sensor is necessary for the programmable soft metamaterial to measure sound pressure level (dB) thresholds (set at 85 dB according to OSHA standards) and to fine-tune the mechanical deformation. This fine-tuning influences the bandgap, band structure, and dispersion curve, achieving partial-to-complete bandgaps for various reduced wave vector directions. In addition, two distinct types of programmable TSMF-lichen must be considered. The first type of the example illustrates the mobility of a smart shade application within a smart home, as demonstrated in **Figure 5K** and **Video S6**. In this configuration, a linear actuator can compress or extend the TSMF-lichen smart shade from right to left (compression) or left to right (decompression/re-expansion) to return it to its original state. In essence, this enables the presence or absence of a TSMF-lichen shade as a window barrier, effectively toggling between null and true existence. In this initial approach, the incident sound wave can interact with the metamaterial from a multitude of angles and directions.

The second approach is based on the orthogonal programmability of soft metamaterials, with the objective of achieving partial-to-complete bandgaps for high-performance sound attenuation. This entails the creation of multiple bandgaps and the manipulation of reduced sound wave vector, as illustrated in dispersion curves in **Figures S2** and **S3**. To achieve this, it is necessary to align the direction of sound wave propagation parallel to the compression and anisotropic scaling in order to realize a complete bandgap. Specifically, the greatest sound attenuation occurs when the incident sound wave direction is perpendicular to the soft TPMS metamaterial, while the lowest performance is observed when the incident sound wave direction is at a 90-degree angle relative to the metamaterial.

As a further illustration, consider a customizable configuration characterized by a blend of soft and hard components (illustrated in **Figure 1b**). In this scenario, each dynamic operational unit is comprised of a single soft TPMS panel situated between two hard TPMS panels, which may be linked to other TPMS elements or a linear actuator arm. To provide a more comprehensive illustration of the second type, we have incorporated **Video S7**, which depicts a multilayered amalgamation of hard and soft TPMS, accompanied by schematic depictions of sound frequency and wave direction. For example, consider a tonal frequency situated at 10,500 Hz. A comprehensive bandgap is evident between the frequency range of 10 kHz and 12.5 kHz, as illustrated in **Figures S2p**. However, upon a frequency shift to 18,200 Hz, as depicted in **Figures** **S2k** and **S2l** with an AS of 75% and 80%, respectively, a complete band range emerges. In order to achieve this outcome, the TSMF-lichen unit cell must be compressed by 20-25% in the X-direction, thereby reducing its dimensions to 75%-80% of their original size. For the sake of clarity, an AS of 75% is selected, which entails a 25% reduction in size for the soft TPMS metamaterial or TMSF-lichen variant.

**S9. Sound Attenuation Metrics and Comparison**

To compare the sound attenuation performances of different graded SLSM (gSLSM) and TMSF-lichen, particularly focusing on the close comparison between Case 3 and Case 5 as shown in **Figure S13**, the integral of the sound attenuation profile with respect to wavelength can be a valuable approach. This approach is particularly useful in the context of broadband noise scenarios, where multiple frequencies across different wavelengths are involved. It provides a comprehensive benchmarking measure.

For the reasons previously stated, Cases 3 and 5 represent two closely performing customized cases. Given the broad interest in sound attenuation performance, particularly in the context of broadband noise, one of the metrics to consider in **Figure S13** is the utilization of discrete integration with respect to the Y-axis (wavelength).


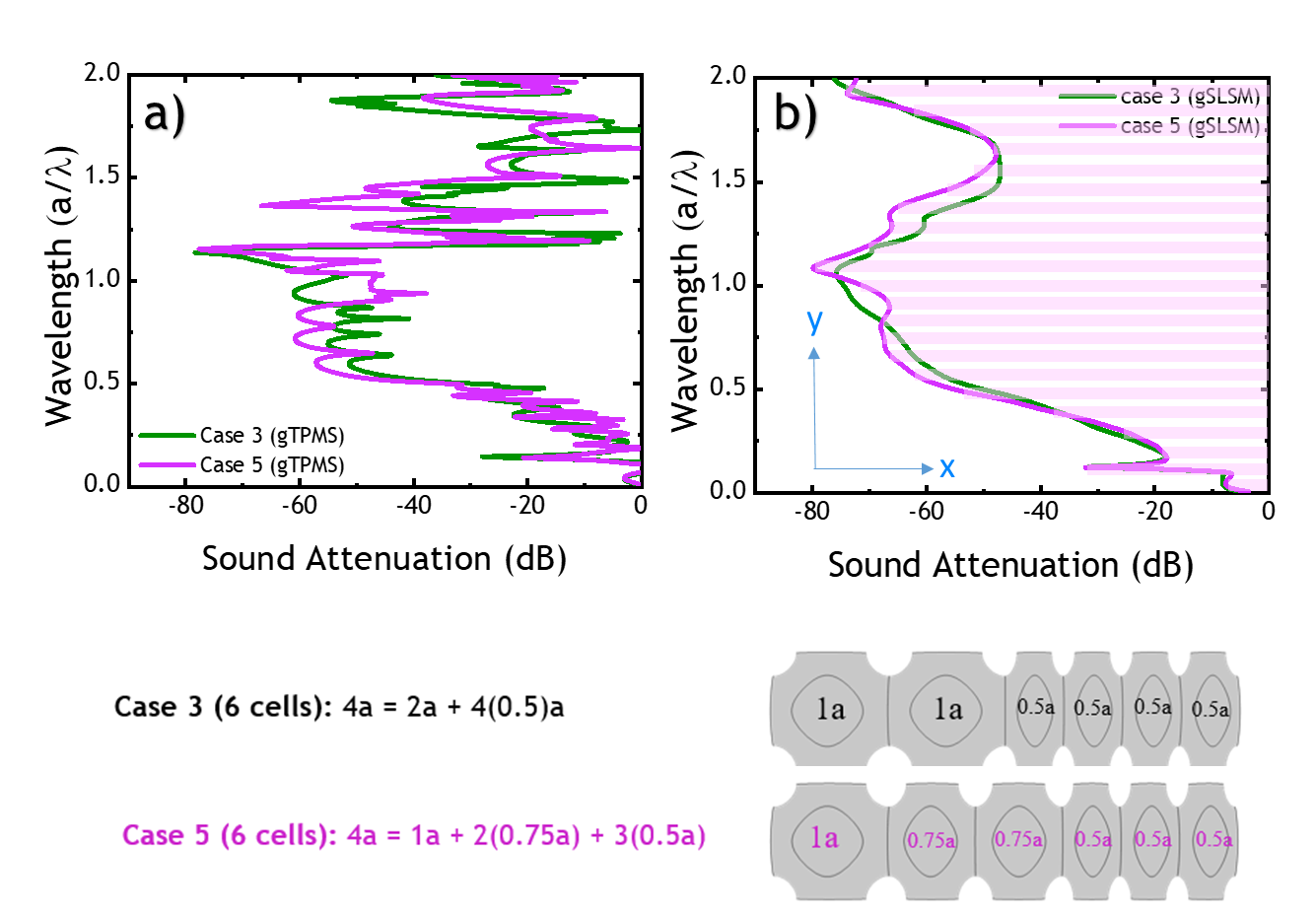


**Figure S13.** Discrete integral to compare broadband wavelength performance for sound attenuation. **a)** gTPMS for case 3 and case5, **b)** gSLSM for case 3 and case 5

In contrast to the analytical approach for a definite integral, computations and applied signal processing are concerned with discrete data and discrete functions of sound attenuation, $F_{SA}\left( \frac{a}{\lambda_{i}^{i+1}} \right)$, which necessitates the discrete integral as a solution for measuring the area for performance comparison. The following integral provides a quantitative method for comparing attenuation. Furthermore, it should be noted that $\lambda_{1}>>\lambda_{n}$.

The definite integral in the analytical solution for **Figure S13b** can be expressed as,

| ${Area}_{SA}=\left\vert\int_{\frac{a}{\lambda_{1}}}^{\frac{a}{\lambda_{n}}} F_{SA}\left( \frac{a}{\lambda} \right)d\left( \frac{a}{\lambda} \right)\text{ } \right\vert=\left\vert\int_{0}^{2} F_{SA}\left( \frac{a}{\lambda} \right)d\left( \frac{a}{\lambda} \right)\text{ } \right\vert$  And general discrete integral for sound attenuation function,$F_{SA}\left( \frac{a}{\lambda_{i}^{i+1}} \right)$, can be expressed as, | (22) |
| --- | --- |
| ${Area}_{SA}=\left\vert\sum_{i=1}^{n} F_{SA}\left( \frac{a}{\lambda_{i}^{i+1}} \right)\Delta\left( \frac{a}{\lambda_{i}^{i+1}} \right)\text{ } \right\vert$  The trapezoidal rule discrete integral formula in applied math can be expressed by the following equation, | (23) |
| ${Area}_{SA}=\left\vert\frac{h}{2}\times\sum_{i=1}^{n} F_{SA}\left( \frac{a}{\lambda_{i}} \right)+F_{SA}\left( \frac{a}{\lambda_{i+1}} \right) \right\vert$  where height, ***h***, in trapezoidal integral for normalized wavelengths, $\frac{a}{\lambda_{i}}$, divided by homogeneous discretization interval, $\boldsymbol{n},$ can be expressed as the following equation. Bear in mind $\lambda_{1}>>\lambda_{n}$, & $\lambda_{i}>\lambda_{i+1}$ for shorter wavelengths at high frequencies. | (24) |
| $h=\Delta\left( \frac{a}{\lambda_{i}^{i+1}} \right)=\frac{a}{\lambda_{i+1}}-\frac{a}{\lambda_{i}}=\frac{a}{n}\left( \frac{1}{\lambda_{n}}-\frac{1}{\lambda_{1}} \right)=\frac{2-0}{n}$ | (25) |
| More area means better sound attenuation performance over total wavelengths.  $\left\vert{Area}_{SA} \right\vert_{case 3}<\left\vert{Area}_{SA} \right\vert_{case 5}$ | (26) |

**Table S2** provides a comprehensive summary of the metric performance for sound attenuation, clearly demonstrating that Case 5 exhibits slightly superior performance to Case 3 across various frequencies and wavelengths in total benchmarking for broadband metrics.

**Table S2.** Discrete integral and area for normalized sound attenuations in decibels (dB)

| **Type** | **Case 1** | **Case 2** | **Case 3** | **Case 4** | **Case 5** |
| --- | --- | --- | --- | --- | --- |
| Area (gTPMS) | 47.68 | 55.38 | 65.29 | 57.61 | 67.86 |
| Area (gSLSM) | 84.57 | 96.77 | 108.65 | 98.45 | 111.53 |

1. [↑](#footnote-ref-1)
